# Oral Cavity Clinical Evaluation in Psychiatric Patients with Eating Disorders: A Case-Control Study

**DOI:** 10.3390/ijerph20064792

**Published:** 2023-03-08

**Authors:** Teresa Szupiany-Janeczek, Krzysztof Rutkowski, Jolanta Pytko-Polończyk

**Affiliations:** 1 Department of Integrated Dentistry, Jagiellonian University Medical College, 31-155 Kraków, Poland; 2 Psychotherapy Department, Jagiellonian University Medical College, 31-008 Kraków, Poland

**Keywords:** eating disorders, oral symptoms of psychiatric diseases, bulimia, anorexia, tooth erosion, oral hygiene, oral health

## Abstract

Bulimia nervosa and anorexia nervosa are not the only disorders the symptoms of which may be present in the oral cavity. The assessment of the clinical condition of patients with eating disorder symptoms was aimed at in this study. The study group consisted of 60 patients with diagnoses from categories F4.xx, F5x.x, and F6x.x ICD-10 (International Classification of Diseases, Tenth Revision). Patients were qualified for the study based on the answers provided in the symptom checklists “O”. An adequate control group was selected. All patients underwent a dental examination, including the assessment of API (aproximal plaque index) and DMF (decayed missing filled index). Studies have shown that patients with eating disorder symptoms were more likely to have dental erosions (in total, 28.81% of cases). The correlation of erosion with the symptoms of eating disorders was demonstrated for several assessed symptoms present in the symptom checklists “O”. Such correlations have not been demonstrated in terms of gingival recession presence. The level of oral hygiene in patients with eating disorders was assessed as sufficient or bad and indicates the need to initiate dental treatment in this group. It is important to correlate the treatment of the underlying mental disease with dental treatment and regular dental checkups.

## 1. Introduction

Patients with eating disorders require indepth diagnostics and multispecialty treatment. In the case of many occurring symptoms, a somatic background has to be ruled out, and both concomitant ailments and the effects of mental disorders need to be diagnosed and treated [1].

Eating disorders are a group of mental illnesses manifested by a distorted perception of oneself, food, unusual eating and hygiene habits, and regurgitating digestive tract contents. The major diseases in this group include anorexia (anorexia nervosa), bulimia (bulimia nervosa), and other unclassified eating disorders. The symptoms of eating disorders may also occur in the course of other mental illnesses. These symptoms can be found in the case of mood disorders, neuroses, and anxiety disorders, as well as in patients suffering from addiction to alcohol or drugs [1,2,3,4].

The role of the dentist in recognizing these clinical problems is important since through a properly conducted interview and a detailed extraoral and intraoral examination, he may be the first physician to recognize the symptoms of an ongoing systemic disease. On the other hand, knowledge of the possible effects of eating disorders in the mouth as the first section of the gastrointestinal and respiratory tracts, and their impact on the local and general condition of the patient, should be part of the education of psychiatrists and psychologists, prompting them to include dental consultation in the management of patients [5].

The social importance of eating disorders increases with the growing role of appearance and changes in beauty patterns in society and the increasing incidence of mental illnesses with eating disorder symptoms in industrialized countries [6,7]. The majority of patients are women (approx. 90%), and the overall incidence of these diseases is 0.5 to 1%. For bulimia alone, these values are 1–5%, and also 40 to 50% of patients with anorexia suffer from bulimia [2,8,9,10].

The causes of eating disorders are not fully understood. Genetic, cultural, and psychological factors appear to affect their presence. The origin is also seen in the early childhood of the patient—problems arise both from eating habits used by caregivers and unpleasant experiences from this period. Some nonspecific risk factors that increase susceptibility to mental problems leading to eating disorders include mental and sexual abuse and personality disorders in the family. People with low self esteem, those with difficulty expressing negative emotions, perfectionists, athletes, and dancers are also prone to these diseases [11]. The genetic factor is also significant in the development of this type of disorder [10].

Anorexia nervosa is characterized by a disturbed body image and a fatal fear of increasing its mass, regardless of the actual weight, which is often far too low (body weight is at least 15% below the expected normal weight for certain ages and heights). Anorexia is more common in women than men. This disorder consists of undertaking numerous, purposeful actions leading to weight loss and maintaining low body weight in the patient. Somatic, metabolic, or neuroendocrine disorders are added with time over the course of the disease [8,12].

Bulimia nervosa is unrestrained attacks of hunger, followed by compensatory behavior, aimed at getting rid of consumed food and avoiding weight gain by taking laxatives, inducing vomiting, using appetite suppressants, and many more [8,12].

Bulimia or anorexia may be the only diagnosis in a patient, however, more often they coexist with other mental disorders such as depression or obsessions, or are part of their symptoms, e.g., when symptoms of eating disorders occur in the course of personality disorders [2,3,8].

Symptoms of nutrition-related abnormalities occur in various forms, e.g., eating disorders, aversion, eating disgust, habitual food regurgitation, digestive disorders with abdominal ailments, and stomachache, flatulence, constipation, or diarrhea [1,6]

Eating disorders can lead to serious metabolic and morphological problems, as well as changes in numerous organs and systems, and sometimes even to a situation that is a threat to a patient’s life [13].

Abnormal eating habits and vomiting can also lead to bradycardia, hypothermia, or hypotension. Anorectic patients with low body weight may have an abnormal or complete loss of menstruation and changes in the ovaries, and both sexes may experience growth retardation and alopecia. In bulimia, gastric contents can be aspirated into the respiratory tract with vomiting, gastric or esophageal ruptures, hypokalemia, arrhythmia, pancreatitis, or drug-induced myopathy, and cardiomyopathy may develop. Patients causing vomiting using fingers may present Russell’s sign, which consists of thickening and redness of the skin of the fingers or hands at the place of contact with the upper incisors [9,14,15,16].

Symptoms of eating disorders in the oral cavity may occur at any stage of the disease and constitute an important indicator in assessing its course, prognosis, and treatment.

The impact of eating disorders on the soft and hard tissues of the mouth depends on both the diet and the duration and intensity of the disease. The occurring symptoms are mostly caused by the irritating effect of vomiting, nutritional deficiencies resulting in metabolic disorders, unusual dietary and hygienic habits, and improper oral hygiene [15,16].

The erosion of enamel and dentin is one of the most frequently observed symptoms within the oral hard tissues [15,17,18]. We observe a phenomenon described as perimylolysis, which is caused by vomiting, gastroesophageal reflux, or belching, the characteristic site for the chemical loss of tooth tissues. The presence of acid of internal origin causes the formation of hard dental tissue defects covering the palatal surfaces of the upper incisors and chewing surfaces of molars and premolars [19]. If the hygiene is not correct and the patient often brushes the teeth immediately after vomiting, the spread of acid and erosion also occurs in other areas of the oral cavity. The supply of external acids by patients in the form of drugs and specifics designed to reduce appetite (e.g., drinking vinegar or sucking a lemon), consumption of large quantities of carbonated drinks and alcohol, or large quantities of energy and isotonic drinks at increased physical activity, are additional factors intensifying this process [20,21,22,23]. The presence of acids of external and internal origin, apart from erosion, results in tooth loss [24]. As a result of significant progress in the process, the anterior teeth may become thinner and shorter and the crowns of the posterior teeth may be shortened until the complete loss of proper anatomical characteristics. The loss of tooth tissue may result in progressive hypersensitivity and, in extreme cases, pulp inflammation, with further consequences in the form of periapical tissue inflammation [19].

A number of scientific studies have confirmed a higher incidence of tooth wear and erosion losses in patients with symptoms of eating disorders, however, there is no consensus on the relationship between the frequency of vomiting and the intensity of observed changes [14].

Increased dental caries in patients with eating disorders is a broad clinical problem. The authors find no correlation between the occurrence of vomiting and the severity of caries. However, there is numerous evidence of more frequent caries-related defects in patients with eating disorder symptoms. This is associated with both a diet rich in simple carbohydrates, a decrease in saliva production, and the avoidance of visits to the dentist for fear of detecting the patient’s mental illness [3,25].

The literature also includes assessments of the impact of eating disorders on the incidence of periodontal disease [25]. The described patients present poor oral hygiene leading to gingivitis and further predisposing to periodontal disease [26]. Vitamin C deficiencies resulting from malnutrition may cause changes in the marginal periodontium and promote gingivitis. Oral cavity dryness associated with deteriorated salivary gland function may affect the condition of the periodontium and oral mucosa [15,27,28]. Nutritional deficiencies can lead to inflammation of the corners of the mouth, tongue mucositis, a burning sensation in the oral cavity, and candidiasis or sores in the mouth. Reduced vitamin supply and iron deficiency anemia can cause mucosal atrophy and an associated burning sensation in the mouth, especially the tongue. As a result of vomiting, there may also be chemical or mechanical (caused by the object used to induce vomiting) mucosal damage [14,27,29].

The occurrence of salivary gland enlargement preceding the belching and vomiting phase is described in the literature. Initially, the swelling of the glands is transient, and it passes into a persistent form with time. This noninflammatory enlargement of the salivary glands, called sialodenosis, is associated with peripheral autonomic neuropathy leading to abnormal secretory activity and enlargement of the salivary glands. These changes may affect small and large salivary glands. In the case of large salivary glands, they are most often bilateral [28].

Initially, both in bulimia and anorexia, there is no quantitative change in saliva production, though there are a number of biochemical deviations in its composition and a decrease in pH [16]. Advanced cases of these disorders lead to a decrease in the flow and amount of saliva, which may be associated with increasing structural changes in the glandular tissue. In addition, xerostomy is a common adverse effect of drugs used in the therapy of individual mental illnesses [3,29].

In patients using dehydrating and laxative agents, there is a variable extent of dehydration and reduced saliva secretion, and the pH decrease associated with the presence of gastric acid may cause pathological changes in the hard palate within the small salivary glands located in this area [14].

Reducing salivation may also increase the susceptibility to both bacterial and fungal infections [28].

Other possible symptoms in the oral cavity include a burning sensation in the mucous membrane, abnormal taste sensation, difficulties in speaking, chewing, and swallowing food, and oral cavity pain of unknown origin. In patients with advanced forms of anorexia, osteopenia, and later osteoporosis may also occur, which may also include the bones of the maxilla and the mandible [3,9,14,29,30].

Aesthetic defects are no less important, but rarely mentioned complications of eating disorders [25].

The main aim of the study was to determine the clinical and microbiological condition of the oral cavity,

The specific objectives of the study included:

1. assessment of the clinical condition of the oral cavity based on the oral hygiene API index and the DMF index;
2. assessment of the presence of dental erosion and gingival recession;
3. check the correlation of eating disorder symptoms in patients with the presence of erosion, recession, and the clinical condition of the oral cavity.

The level of oral hygiene in patients with eating disorders was assessed as sufficient or bad and it did not differ from the control group.

General symptoms of mental-related eating disorders were conducive to tooth erosion development.

## 2. Materials and Methods

### 2.1. General Methodology

The study was carried out in two stages.

In the first stage, among patients of the Day Hospital for Neurotic and Behavioral Disorders of the Psychotherapy Department in the University Hospital in Krakow, the study included patients with diagnoses from categories F4.xx, F5x.x, F6x.x of International Classification of Diseases, Tenth Revision (ICD-10), accompanied by the symptoms of eating disorders. Patients were qualified for the study based on the answers provided in the KO “O” symptom questionnaire, the so-called symptom checklists “O” [31,32].

From among 138 symptoms present in this questionnaire, 12 potential symptoms of various types of eating disorders were selected. Patients who reported the occurrence of at least one of the 12 analyzed symptoms, regardless of their severity, were analyzed. The questionnaires were evaluated regardless of the patient’s primary diagnosis.

The study included patients who had the following symptoms listed in the symptom checklists “O” [31,32]:

1. No. 3: Choking in the throat, a sensation of having a “ball in the throat”;
2. No. 9: Vomiting in situations of nervousness;
3. No. 49: Oral dryness;
4. No. 54: Lack of appetite;
5. No. 57: Constant attention to body functions—heart rate, pulse, digestion, etc.;
6. No. 59: Attacks of hunger—e.g., a necessity to eat at night;
7. No. 69: Diarrheas;
8. No. 74: Constipations;
9. No. 94: Excessive salivation in the mouth;
10. No. 98: Excessive thirst;
11. No. 131: Burning sensation in the esophagus, heartburn;
12. No. 136: Nausea, queasiness.

The study included 60 patients of the Psychotherapy Department in the University Hospital, a control group with the same number was selected similar in terms of gender and age. It consisted of patients of the University Dental Clinic, who, during a medical interview denied the presence of any mental illness presently and in the past. In the second stage, patients who qualified for the study group were admitted to the University Dental Clinic, where oral clinical examination and additional examinations were carried out.

### 2.2. Clinical Examination Methodology

Each patient underwent a dental examination according to generally accepted principles. Next, a detailed extraoral and intraoral examination was performed. In addition, periodontal and oral hygiene examinations were carried out using a WHO periodontometer. The oral examination was assessed based on the following indicators:

1. API index;
2. DMF index.

Lange’s API (aproximal plaque index) assesses the presence of bacterial plaque in interdental spaces [32]. It is calculated as the percentage ratio of the space with the current bacterial plate to all the spaces examined. The presence of the plaque was checked using a WHO periodontal probe. The index value is determined as a percentage, and the results are interpreted as follows:

API 70–100%—insufficient oral hygiene;

API 40–69%—sufficient hygiene, but improvement is recommended;

API 25–39%—quite good oral hygiene;

API < 25%—optimal oral hygiene;

DMF—decayed missing filled indicator is an index of the intensity of the dental caries process, at the same time determining the incidence of this disease [33]. It consists of the assessment of the following components:

D—teeth with one or several primary or secondary caries defects and with temporary dressing; M—teeth lost or removed due to caries; F—teeth with one or more fillings.

The DMF index is the sum of the individual components: DMF = D + M + F. A number of DMF greater than 0 indicates that the person is or has been affected by caries. This is indicated by the presence of only one filling (D = 0, M = 0, F = 1 so DMF = 1). On the other hand, the DMF number expressing the sum of these three values does not provide clear information about the tooth condition. In extreme cases, both toothless people, as well as those who have all the teeth, but each one of them filled, will have a DMF score of 32. Due to that, both the DMF sum and the values of individual components of the index and their impact on other parameters describing the condition of the oral cavity were analyzed. For every patient and every component, the DMF index can score from 1 to 32, giving too numerous options. So for the purpose of statistical analysis, the DMF index values were grouped into ranges (Table 1).

During the clinical examination of the patients, attention was also paid to the presence of erosive defects and gingival recessions in the 0–1 system (a single defect or recession, regardless of the number of defects/recessions and their severity, meant the patient was assigned to the group affected by this problem).

The clinical examination was supplemented by a patient’s orthopantomographic scan, allowing the assessment of alveolar processes of the jaws, and the alveolar part of the mandible, along with teeth.

Each patient had photographic documentation prepared, including photos of a smile, closed teeth with retraction, and upper and lower arch with retraction.

### 2.3. Statistical Test Methodology

The results of clinical tests were systematized in a Microsoft Excel table.

The collected data was first developed using descriptive statistics tools and variable distributions (Chi-square test, F = Fisher’s exact test, Mann–Whitney test depending on the situation). When testing hypotheses, the significance level was adopted at α = 0.05.

Software R, version 3.4.3, was used for the analysis. 

(R Core Team (2017). R: A language and environment for statistical computing. R Foundation for Statistical Computing, Vienna, Austria.

URL https://www.R-project.org/ accessed on 18 September 2017

Jagiellonian University Bioethics Committee approval and code:

KBET/65/B/2012 from 22 March 2012

## 3. Results

### 3.1. Structure of the Studied Group

The study included 60 patients, and another 60 people constituted a similar control group in terms of sex and age. Women constituted 76.27% of the study group and 75% of the control group. The ages of the women in the analyzed groups had a normal distribution (*p* > 0.05 from the Shapiro–Wilk test), so the analysis was carried out using the Student’s *t*-test. On the other hand, the age of men did not have a normal distribution in the analyzed groups (*p* < 0.05 from the Shapiro–Wilk test), so the analysis was carried out using the Mann–Whitney test. The mean age of women in the study group was 29.76 (SD 5.26), with an age range between 20 and 43 years, in the control group: 29.51 (SD 5.28), with an age range between 20 and 43 years, and 33.29 among men (study group), with an age range between 25 and 52 years (SD 6.54), and 34.27 (control group), with an age range between 23 and 56 years (SD 9.01). The age structure (mean, median, and quantile values) for women and men are presented in Table 2.

### 3.2. API Index

**Figure 1:**
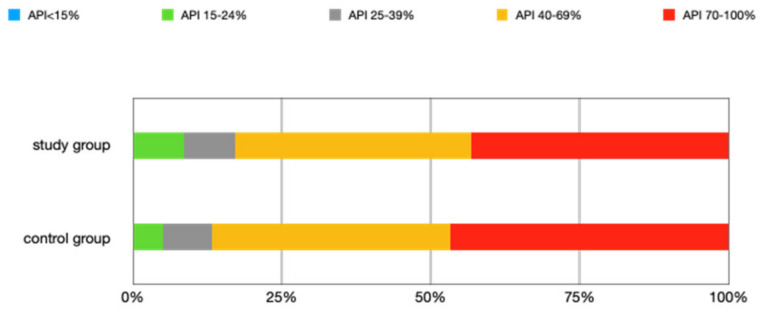
The API score is pictured according to interpretation values. Note—there were no patients with an API index below 15%.

**Figure 2:**
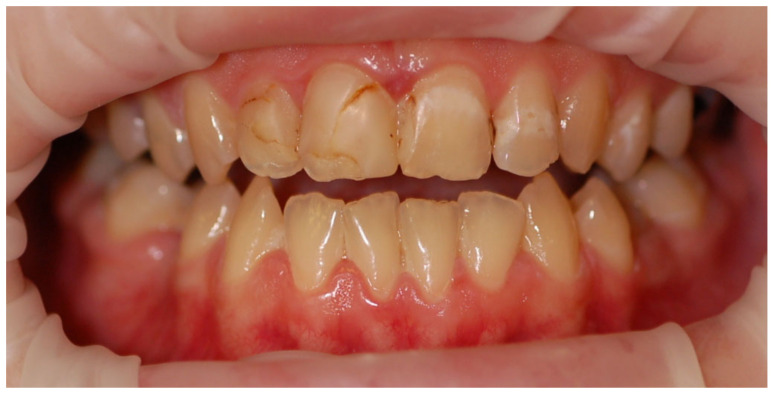
Woman, 23 y.o. Clinical situation, API = 100%. Dental Biofilm-induced Gingivitis. Leaking restorations.

**Figure 3:**
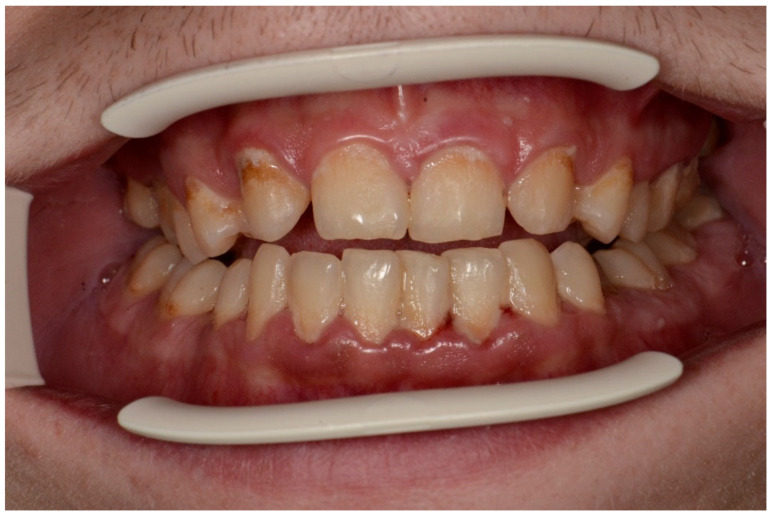
Man, 28 y.o. API = 100%. Calculus and dental plaque deposits. Dental Biofilm-induced Gingivitis. Teeth discoloration and superficial caries lesions.

**Figure 4:**
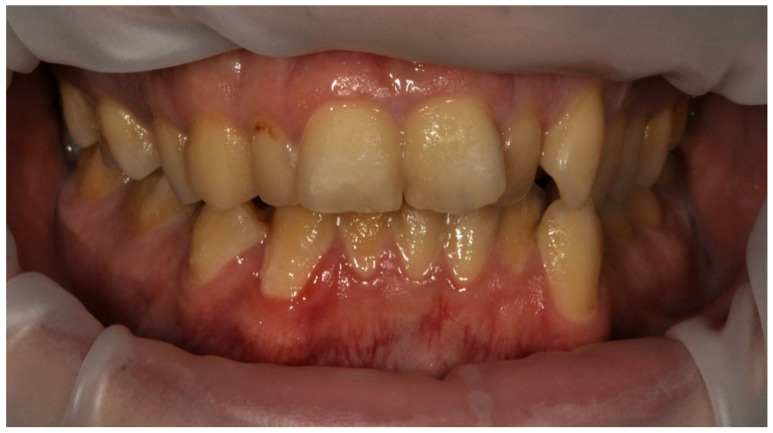
Man, 42 y.o. API = 100%. The patient refused to use professional oral hygiene products and used soap to brush his teeth. Dental plaque deposits. Dental Biofilm-induced Gingivitis.

The mean API score among the subjects was 63.47% (SD = 22.78) and ranged between 22 and 100%. The mean value of the index for the control group was 68.75% (SD 21.46) and ranged from 19 to 100%. The *p* > 0.05, therefore the study and control groups did not differ significantly from the API value. The median value of the API index for the study group was 65 and 68.5 for the control group. Table 3 and Figure 1 present the API score and the API score pictured according to interpretation values. Figure 2, Figure 3 and Figure 4 show the clinical situation of the participants. The API values did not have a normal distribution in the analyzed groups (*p* < 0.05 from the Shapiro–Wilk test), so the analysis was carried out using the Mann–Whitney test.

### 3.3. DMF Index

The median value of the DMF Index in the study group was 17 (12–19) and 17 (14–19.25) in the control group (Table 4).

**Figure 5:**
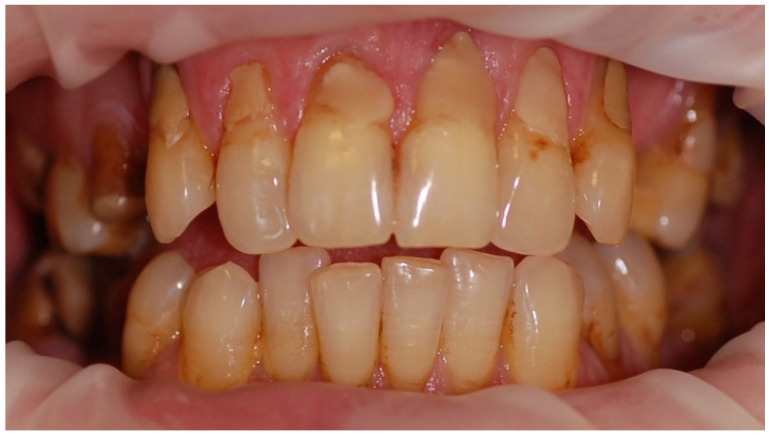
Man, 52 y.o. Clinical situation presenting very high DMF value.

All *p* > 0.05, so the study and control groups did not differ significantly in terms of the value of the DMF index or any of its components. It is noteworthy that on average in the study and control group, the patients had more than four caries-related defects and at least one tooth was removed. The patient’s clinical situation with a very high DMF value is shown in Figure 5.

The detailed distribution of the DMF Index values and its components in the examined and control group are presented in Table 5. Additional analysis also showed that the values of the DMF index and its individual components did not significantly depend on sex in both the examined and control groups.

The DMF values did not have a normal distribution in the analyzed groups (*p* < 0.05 from the Shapiro–Wilk test), so the analysis was carried out using the Mann–Whitney test.

### 3.4. Recession

In the study group, a recession was found in 25.42% of patients and the control group in 31.67%.

The *p* > 0.05, so the study and control groups do not differ significantly in the occurrence of recessions (Chi-square test) (Table 6).

### 3.5. Erosion

The presence of erosive defects was found in a total of 28.81% of patients in the study group compared to 3.33% in the control group.

The *p* < 0.05, so the study and control groups differ significantly in the occurrence of erosion: in the study group they occur more frequently (Chi-square test) (Table 7).

These relationships were also confirmed in women, where 35.56% of the patients in the study group had at least one erosive defect (compared to the control group, where the presence of these defects was found in 4.44% of the subjects). The *p* < 0.05, so the study and control groups differ significantly in the occurrence of erosion; in the study group, they occur more often. In the case of men, no one in the control group presented symptoms of erosion, and in the study group, it occurred in 7.14% of patients. The *p* > 0.05, so the study and control groups do not differ significantly in the occurrence of erosion.

**Figure 6:**
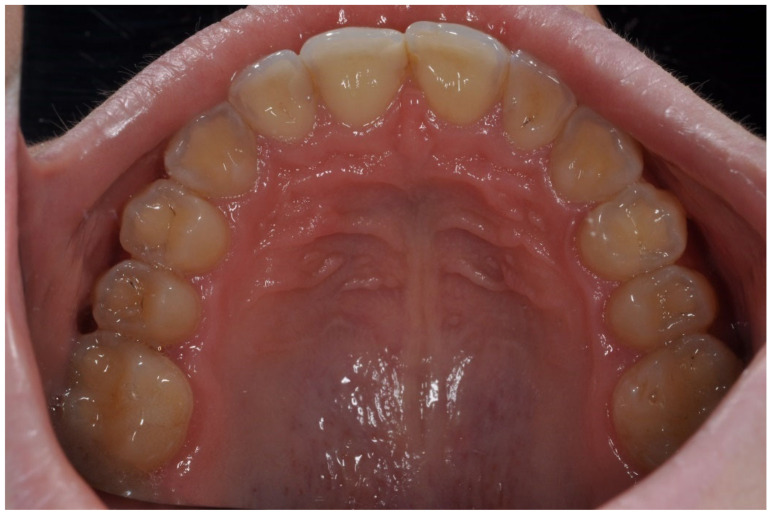
Woman, 26 y.o. Perimylolysis in a patient with the main diagnosis: mixed neurotic disorder, showing symptoms of eating disorders.

**Figure 7:**
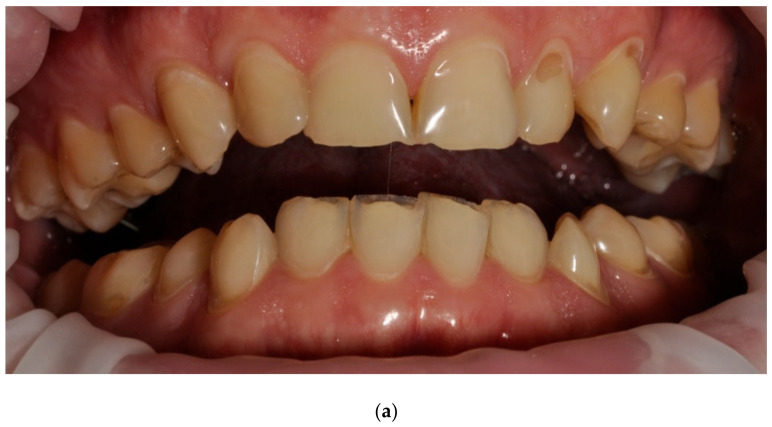

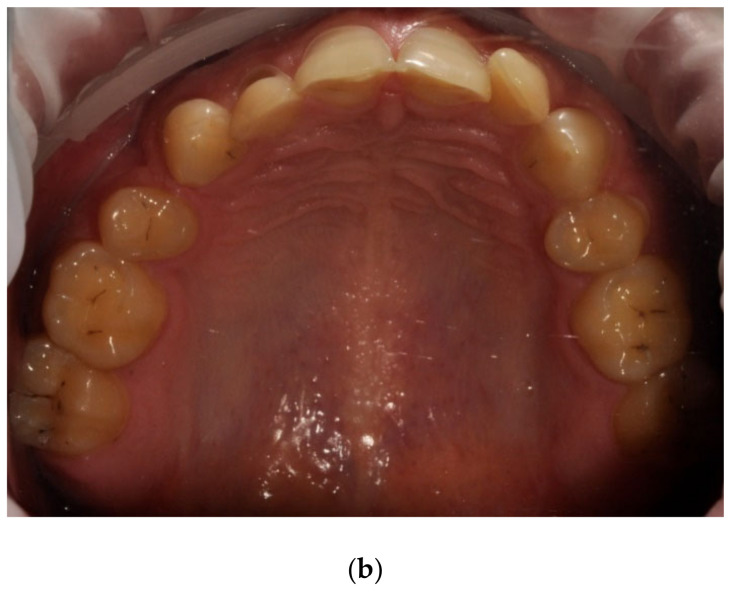
(**a**,**b**) Man, 33 y.o. Advanced internal and external erosion. The patient reported regular vomiting and consumption of approximately 6 L of Coca-Cola per day over a period of more than 6 months—front teeth and upper arch.

**Figure 8:**
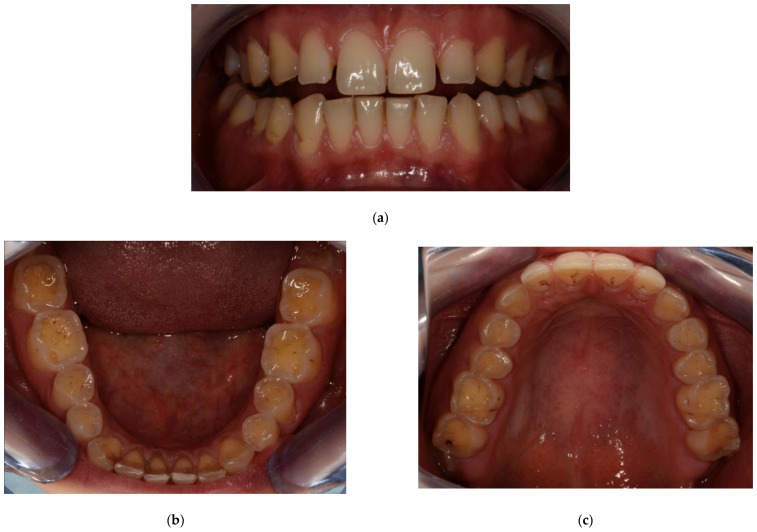
(**a**–**c**) Woman, 29 y.o. Patient with advanced erosion and tooth wear—front view and upper and lower arches.

Examples of erosive defects occurring in the examined patients are presented in Figure 6, Figure 7a,b and Figure 8a–c.

### 3.6. Distribution between the Presence of Any of the Symptoms of Eating Disorders Constituting Inclusion in the Study and the Presence of Recession and Erosion

1. Correlation with recession

The occurrence of symptoms from the symptom checklists “O” and recession are not related (*p* > 0.05 for the whole group and men and women separately). Statistical analysis was performed using the Chi-square test and Fisher’s exact test (low values were expected).

2. Correlation with erosion

*p* < 0.05 for symptoms:

- No. 57: Constant attention to body functions—heart rate, pulse, digestion, etc.;
- No. 69: Diarrheas;
- No. 74: Constipations;
- No. 98: Excessive thirst;
- No. 136: Nausea, queasiness.

This means that these symptoms are associated with erosion. Erosion is more common in patients indicating these symptoms of symptom checklists “O” (Table 8). Statistical analysis was performed using the Chi-square test and Fisher’s exact test (low values were expected). Please note that percentages do not add up to 100% as it was a multiple-choice statistical question. Each patient could point out any number of symptoms fitting their problem.

## 4. Discussion

### 4.1. Oral Hygiene and Dental Treatment Needs of the Subjects

The assessment of oral hygiene was based on the API hygiene index. Many researchers emphasize the lack of statistically significant differences in the values of this index in patients with eating disorder symptoms compared to the control group [3,25,30]. This study also indicated the lack of this relationship, showing additionally the poor oral hygiene of all patients. Half of the patients in the study group and half of the control group had an API value above 65 and 68.5 (median value for the study and control group). Index values in the range of 40–69% mean sufficient hygiene and indicate the need to improve it, and values above 70% mean insufficient hygiene. No studies have found an API value below 15% in anyone, and 10.17% of patients with eating disorders and 5% of control people had optimal oral hygiene (API below 25%).

Many researchers indicate the lack of significant differences in the group of patients, compared to people without eating disorders, also in relation to other indicators for assessing the condition of the oral cavity, such as PL (Plaque Index) or PBI (Periodontal Bleeding Index) [25,30]. The significant discrepancies in the presented level of oral hygiene in patients are noteworthy. Individual examinations indicate better hygiene compared to the control group, which is probably associated with frequent brushing, e.g., after vomiting [20,30]. However, other studies found worse oral hygiene compared to the control group and a larger amount of residual plaque [25]. The differences probably result from the disharmony of the analyzed groups in terms of age, origin, property status, and in most cases lack of reference to the nature and course of the subject’s mental disorder.

### 4.2. Teeth Condition

#### 4.2.1. DMF

DMF was used to assess the condition of patients’ teeth. The study and control groups did not differ significantly in terms of the value of the DMF index or with any of its components both in total and by sex. In the literature, you can find studies that confirm the lack of correlation between the value of the DMF index and the symptoms of eating disorders [3,30]. This indicator is usually considered as a sum for patients, without a detailed list of components. It is noteworthy that, on average, in the study and control group, the patients had more than four caries-related defects, and at least one tooth was removed due to caries. The average DMF value among the examined group was 16.1 (17.4 in the control group). This result is consistent with the observations from the WHO report from 2010, which places Poland among the countries with a high severity of caries [34]. Patients with eating disorder symptoms, when compared to the control group and the mean for the Polish population, do not have significantly higher levels of caries.

#### 4.2.2. Erosion

The presence of erosive defects was found in a total of 28.81% of the study group, compared to 3.33% in the control group, which provides a statistically significant difference. In a detailed study by gender, 35.56% of women experienced erosion (this value was 7.15% for men). These results correspond to the data available in the literature [20,21,24,30]. The occurrence of erosion as a result of the supply of acids from the outside and the presence of acids of internal origin is a common and widely described phenomenon. Most often, however, the study group is limited to women, hence it is not entirely possible to compare the obtained results. The increasing incidence of mental disorders with eating disorders and the increasing percentage of men among patients prove the sense of analyzing a study group including both women and men. The available literature indicates the most frequent location of erosion-related cavities on the palatal surfaces of superior incisal teeth and the occlusal surfaces of inferior molar teeth. In the case of erosion of external origin, these are the labial surfaces of the incisors and upper posterior teeth. According to the literature, visible signs of erosion appear after 6 months of regular vomiting, providing a characteristic clinical picture of this process [16,20,30]. A detailed analysis of the location or stage of erosion-related losses was not assessed in this study. Their presence was studied according to a system in which zero meant no losses, and one meant at least one erosion loss, whereas clinical examinations found numerous examples of erosion-related losses of different origins, the representation of which was included in the results of the study. The appearance and location of the erosions were found to correspond to the data in the literature. Further analysis certainly requires an assessment of the severity of lesions depending on the course of the disease and their location, resulting from the origin of the active acid.

#### 4.2.3. Recession

Available literature confirms the higher incidence of recession in anorectic patients [25,30]. Frequent and often incorrect brushing after vomiting leads to and/or intensifies this process in patients with other types of eating disorders [20]. The conducted study demonstrated a high percentage of recession (in 25.42% of patients, and 31.67% in the control group), however, there were no statistically significant differences showing the impact of eating disorder symptoms on the incidence of recession in the analyzed group of patients.

### 4.3. The Relationship between API, DMF Components and Erosive Defects and Gingival Recession

Significant differences were found between women and men in the study group. Women had lower D values and erosions. The control group showed no differences regarding the association of the foregoing parameters. The results described in terms of lower values of the D index in women compared to men correspond with data on the general population (without eating disorders and other additional diseases) [3,30,34]. Most of the available publications studying the DMF index and erosion describe patients with specific diagnoses such as bulimia and anorexia and study only women. On the other hand, analyses of various symptoms of eating disorders in women and men from the perspective of psychiatrists give an opinion on the possibility, nature, and frequency of the effect of symptoms on the oral cavity condition. Nevertheless, they do not provide a detailed list of described symptoms and indexes for the assessment of the oral cavity condition [3,30].

### 4.4. The Effect of Individual Symptoms of Eating Disorders on the Presence of Recession and Erosion

The study did not show any association of any of the 12 analyzed symptoms with the presence of recession. On the other hand, the presence of several relationships deserves attention in the analysis of erosive defects. In women, erosion was more common when they indicated that they were suffering from diarrhea (symptom no. 69), thirst (symptom no. 98), burning sensation in the esophagus, heartburn (symptom no. 131), and nausea and nausea (symptom no. 136). In men, erosions occurred correlated with symptom no. 57—constant attention to body functions—heart rate, pulse, digestion, etc. The significance of these symptoms for the occurrence of erosive defects seems fairly obvious in most of them, and in the case of symptoms no. 57 and no. 69, it requires deeper analysis and further research. Due to the pioneering nature of the comparison, it was not possible to find literature related to this topic. It seems that the assessment of the time of symptom onset and their activity and intensity can be an important clinical translation, thanks to the observation of erosion foci, in terms of the data from the medical history and symptom questionnaires.

## 5. Summary

This study was treated extensively, without limiting it to the analysis of patients with a diagnosis indicating the necessity of oral cavity examination due to the possible manifestation of individual diseases in this region, as described in the literature. For bulimia, anorexia, and other unclassified eating disorders, the relationship and effect on oral health are well understood and studied. Therefore, this study focuses on patients with different primary diagnoses, including those outside the group of eating disorders, and inclusion in the study was determined by symptoms, rather than the diagnosis having been made. The use of the symptom checklists “O” showed the significant effect of the symptoms on the condition of the oral cavity and thus expanded the group of patients requiring referral for additional dental tests. For dentists, the results of this study mean that there is a broader group of patients to whom, during a thorough examination of the oral cavity, attention should be paid to potential symptoms of eating disorders manifesting themselves in the oral cavity. Reports from the literature confirm the importance of specialist education, extensive oral diagnostics, and cooperation of physicians from many fields in the diagnosis, treatment, and secondary prevention of many mental diseases [35,36,37]. Based on the results of the foregoing study and literature analysis, a list of recommendations for the care of a patient with eating disorder symptoms was formulated [4,11,12,37,38]:

- Follow-up visits at the initial stage of treatment should take place every three months;
- It is advisable to perform scans of the oral cavity, which, in addition to a valuable element of medical documentation, facilitate communication with the patient;
- Frequent hygienization procedures should be accompanied by documenting the therapy progress in relation to changes in the hygiene index values;
- Fluoride rinses, pastes with high fluoride content, and frequent professional fluoridation treatments by various methods should be recommended to patients with high-intensity of caries, hypersensitivity, and erosion;
- Patients after vomiting should be advised to rinse their mouth with water and avoid brushing their teeth immediately after this episode. In addition, they should clean their tongue, since it may contain acidic content, even after rinsing their mouth and brushing their teeth;
- Encourage patients to drink only water frequently, and use a “straw” for other drinks;
- Cooperation with a psychiatrist and psychologist, joint assessment of treatment progress in terms of the general disease and the moment of starting to treat the effects of eating disorder symptoms, e.g., reconstruction of lost tooth tissues.

## 6. Conclusions

In patients with eating disorders, the level of oral hygiene (based on API values) is sufficient or poor, which indicates the need to initiate dental treatment in this group of patients.

The presence of dental erosion correlates with the individual general symptoms of eating disorders. Such correlations have not been demonstrated in terms of gingival recession presence.

In the case of psychiatric patients with eating disorder symptoms (also diagnosed outside the group of typical disorders), it is important to correlate the treatment of the underlying disease with dental treatment.

## Figures and Tables

**Table 1:** DMF index values grouped into presented ranges to facilitate statistical analysis.

Index or Its Component	Ranges	Index or Its Component	Ranges
DMF	0–4	D, M, and F index	0
	5–9		1–3
	10–14		4–7
	15–19		8–11
	>19		>11

**Table 2:** The age structure (mean, median, and quantile values) for women and men.

Sex	Group	N	Mean	SD	Median	Min	Max	Q1	Q3	*p* *
Female	Study	45	29.76	5.26	29	20	43	25	33	0.826
	Control	45	29.51	5.28	29	20	43	26	32	(*t*)
Male	Study	14	33.29	6.54	32.5	25	52	29.25	35	0.983
	Control	15	34.27	9.01	32	23	56	28.5	35.5	(MW)

* *t*—*t*-Student test, MW—Mann–Whitney test.

**Table 3:** The API score (median and quartiles) for the study and control group. Note—there were no patients with an API index below 15%.

Index	Group	N	Median	Min	Max	Q1	Q3	*p* *
API [%]	Study	59	65	22	100	46.5	77.5	0.211
	Control	60	68.5	19	100	58.5	83.5	

* Mann-Whitney test.

**Table 4:** DMF index for women and men joined.

Index	Group	N	Median	Min	Max	Q1	Q3	*p* *
DMF	Study	59	17	4	32	12	19	0.278
	Control	60	17	6	26	14	19.25	
D	Study	59	3	0	22	2	6	0.094
	Control	60	4	0	13	2.75	6	
M	Study	59	0	0	23	0	2.5	0.421
	Control	60	0	0	12	0	2.25	
F	Study	59	9	2	18	7	13	0.09
	Control	60	11	6	17	9	13	

* Mann-Whitney test.

**Table 5:** The detailed distribution of DMF index values and its components.

DMF Index Values and Its Components		Study Group (N = 59)		Control Group (N = 60)	
		n	%	n	%
DMF	0–4	1	1.69%	0	0.00%
	5–9	7	11.86%	2	3.33%
	10–14	17	28.81%	14	23.33%
	15–19	20	33.90%	29	48.33%
	>19	14	23.73%	15	25.00%
D	0	5	8.47%	4	6.67%
	1–3	28	47.46%	17	28.33%
	4–7	17	28.81%	31	51.67%
	8–11	7	11.86%	7	11.67%
	>11	2	3.39%	1	1.67%
M	0	31	52.54%	37	61.67%
	1–3	17	28.81%	13	21.67%
	4–7	9	15.25%	5	8.33%
	8–11	1	1.69%	4	6.67%
	>11	1	1.69%	1	1.67%
F	0	0	0.00%	0	0.00%
	1–3	2	3.39%	0	0.00%
	4–7	15	25.42%	6	10.00%
	8–11	19	32.20%	28	46.67%
	>11	23	38.98%	26	43.33%

**Table 6:** Percentage of gingival recession in the entire study group.

Clinical Symptom	Study Group (N = 59)		Control Group (N = 60)		*p* *
	n	%	n	%	
Recession	15	25.42%	19	31.67%	0.582
No recession	44	74.58%	41	68.33%	

* Chi-square test.

**Table 7:** Occurrence of erosive defects in the study group of patients.

Clinical Symptom	Study Group (N = 59)		Control Group (N = 60)		*p* *
	n	%	n	%	
Erosion	17	28.81%	2	3.33%	<0.001

* Chi-square test.

**Table 8:** The results of the distribution study between the presence of any of the analyzed symptoms and the presence of erosion (symptoms with correlation found were marked in bold text).

Check List “O” Symptoms	Erosion (N = 19)		No Erosion (N = 100)		*p*
	n	% *	n	% *	
Symptom No.3	7	36.84%	17	17.00%	0.063
Symptom No. 9	2	10.53%	4	4.00%	0.244
Symptom No.49	6	31.58%	17	17.00%	0.201
Symptom No. 54	6	31.58%	19	19.00%	0.228
**Symptom No. 57**	2	10.53%	0	0.00%	**0.024**
Symptom No. 59	5	26.32%	12	12.00%	0.146
**Symptom No. 69**	6	31.58%	9	9.00%	**0.015**
**Symptom No. 74**	6	31.58%	11	11.00%	**0.03**
Symptom No. 94	1	5.26%	3	3.00%	0.506
**Symptom No. 98**	10	52.63%	17	17.00%	**0.002**
Symptom No. 131	8	42.11%	22	22.00%	0.084
**Symptom No. 136**	12	63.16%	19	19.00%	**<0.001**

* Chi-square test, F = Fisher’s exact test (low values expected).

## Data Availability

Data available on request due to restrictions e.g., privacy or ethical. The data presented in this study are available on request from the corresponding author. The data are not publicly available due to privacy issues.

## Abbreviations

API	Aproximal Plaque Index (according to Lange)
ICD-10	International Classification of Diseases, Tenth Revision
MAX	maximum value
MIN	minimum value
DMF	Decayed Missing Filled Index
Q1	first quantile
Q3	third quantile
SD	standard deviation
WHO	World Health Organization
